# Porous Cage Macro-Topography Improves Early Fusion Rates in Anterior Cervical Discectomy and Fusion

**DOI:** 10.1155/2024/8452050

**Published:** 2024-03-14

**Authors:** Gregory M. Malham, Dean T. Biddau, Jordan P. Laggoune, Charlie R. Faulks, William R. Walsh, Yi Yuen Wang

**Affiliations:** ^1^Neuroscience Institute, Epworth Hospital, Richmond, Melbourne, Australia; ^2^Department of Surgery, The University of Melbourne, Melbourne, VIC, Australia; ^3^Spine Surgery Research, Swinburne University of Technology, Melbourne, VIC, Australia; ^4^School of Biomedical Sciences, Queensland University of Technology, Brisbane, QLD, Australia; ^5^Surgical & Orthopaedic Research Laboratories (SORL), UNSW Sydney, Prince of Wales Clinical School, Prince of Wales Hospital, Sydney, Australia

## Abstract

**Objectives:**

Anterior cervical discectomy and fusion (ACDF) aims to improve pain, relieve neural compression, achieve rapid solid bony arthrodesis, and restore cervical alignment. Bony fusion occurs as early as 3 months and up to 24 months after ACDF. The correlations between bony fusion and clinical outcomes after ACDF remain unclear. Macro-topographic and porous features have been introduced to interbody cage technology, aiming to improve the strength of the bone-implant interface to promote early fusion. In this study, we aimed to compare clinical outcomes and CT-evaluated fusion rates in patients undergoing ACDF using one of two different interbody cages: traditional NanoMetalene™ (NM) cages and NM cages with machined porous features (NMRT).

**Methods:**

This was a prospective, observational, nonrandomised, cohort study of consecutive patients undergoing ACDF. The NM cage cohort was enrolled first, then the NMRT cohort second. The visual analogue scale, neck disability index, and 12-item Short Form Survey scores were evaluated preoperatively and at 6 weeks, 3 months, and 6 months. The minimum clinical follow-up period was 12 months. Plain radiographs were obtained on postoperative day 2 to assess instrumentation positioning, and computed tomography (CT) was performed at 3 and 6 months postoperatively to assess interbody fusion (Bridwell grade).

**Results:**

Eighty-nine (52% male) patients with a mean age of 62 ± 10.5 years were included in this study. Forty-one patients received NM cages, and 48 received NMRT cages. All clinical outcomes improved significantly from baseline to 6 months. By 3 months, the NMRT group had significantly higher CT fusion rates than the NM group (79% vs 56%, *p*=0.02). By 6 months, there were no significant differences in fusion rates between the NMRT and NM groups (83% vs 78%, *p*=0.69). The mean Bridwell grade at 6 months was 1.4 ± 0.7 in the NMRT group and 1.8 ± 1.0 in the NM group (*p*=0.08).

**Conclusions:**

With both NM and NMRT cages, serial improvements in postoperative clinical outcomes were associated with fusion progression on CT. NMRT cages demonstrated significantly better fusion at 3 months and a trend toward higher quality of fusion at 6 months compared with NM cages, suggesting earlier cage integration with NMRT. An early 3-month postoperative CT is adequate for fusion assessment in almost 80% of patients undergoing ACDF with an NMRT cage, permitting an earlier return to activity.

## 1. Introduction

Anterior cervical discectomy and fusion (ACDF) is the most common operation for treating cervical degenerative disc disease, radiculopathy, myelopathy, instability, and deformity [1, 2]. The goals of this surgery are to improve pain, relieve neural compression, achieve rapid solid bony arthrodesis, and restore cervical alignment. Fusion between adjacent vertebrae is achieved by on-growth and in-growth of vertebral endplate bone to both the interbody cage and graft [3]. Interbody cages are usually constructed of polyether ether ketone (PEEK) or titanium (Ti), and their aperture is filled with autograft, allograft, or a variety of synthetic bone substitutes [4]. Anterior plating, either separate or integrated with the cage, increases fusion and lowers subsidence rates [5, 6]. The fusion rate of ACDF is over 90%, with most reoperations performed for symptomatic pseudoarthrosis, which occurs in up to 14% of patients with ACDF after 12 months [7–10].

Traditionally, bony fusion is assessed by visualisation of bridging trabecular bone on plain radiographs or the absence of motion on flexion/extension x-rays [9]. Use of radiographs alone, however, may underestimate the true incidence of pseudoarthrosis, whereas computed tomography (CT) provides excellent qualitative and quantitative measurements of interbody bone, with high interobserver reliability [11, 12]. Bony fusion occurs as early as 3 months and up to 24 months after ACDF, with most studies reporting fusion rates at 6 months postoperatively [9, 13]. Whether clinical outcomes correlate with bony fusion after ACDF remains unclear, as some studies reported no association whereas others reported a positive correlation [14–20].

The overall performance of any implantable device is influenced by the choice of material, the design of the device, and the surface of the material, all of which play a role in its biomechanical properties and the biological host-implant interface. The radiolucent nature of PEEK facilitates postoperative assessment of fusion by radiographs or CT, in contrast to solid and/or porous Ti cages [21]. In addition, PEEK is hydrophobic, whereas Ti is hydrophilic, which can influence protein adsorption to the device surface [22]. Combining the mechanical properties and radiolucency of the PEEK modulus with the surface benefits of Ti endplate osseointegration appears advantageous.

Technologies have been developed to apply Ti over all surfaces of PEEK cages using molecular bonding, resulting in a continuous layer that is thin enough to not be visible on radiographs or CT images but more resistant to the flaking seen with Ti plasma sprays [23]. One such technology, NanoMetalene™ (SeaSpine), has been previously studied in large animal models, in which it produced encouraging results [3, 24]. NanoMetalene (NM) technology creates a pure Ti layer that is molecularly bonded to the surface of PEEK rather than acting as a coating, and it has been shown to facilitate bone on-growth [24]. The Ti layer overcomes the shortcomings of the hydrophobic nature of PEEK by providing Ti at the interface with host bone, while maintaining a radiolucent implant and the mechanical properties of PEEK that are more favourable to bone, compared with Ti alloys.

Recently, there has been an introduction of various technologies for interbody cages that have incorporated porous features, such as 3D-Ti and porous PEEK, as well as macro-topography features, all aimed at improving the strength of the bone-implant interface to promote early fusion. Interbody cages with these features have been evaluated in various animal models; however, additional variables existed in these studies, preventing definitive conclusion about the effects of porosity [25]. To evaluate the benefits of these porous features, it would be advantageous to perform a controlled clinical study of cages with and without porous features, with all other variables (material, cage geometry/footprint, and bone graft) controlled.

In this study, we aimed to compare clinical outcomes and CT-evaluated fusion rates in patients undergoing ACDF using one of two different interbody cages, which were produced by the same company and differed only according to the presence or absence of machined porous features to promote bone in-growth and interlocking. This controlled study design allowed us to isolate the effects of endplate interlocking features and directly evaluate whether there were early clinical or radiologic benefits.

## 2. Methods

### 2.1. Study Design and Patient Population

This was a prospective, observational cohort study of consecutive patients undergoing ACDF from March 2020 to June 2021. All operations were performed by two senior spinal fellowship-trained neurosurgeons using the same surgical techniques (GMM & YYW). Treatment groups were not randomised. This was a nonblinded study. The traditional NM cage cohort was enrolled first, then the NM cage with machined porous features (NMRT) cohort second. The minimum follow-up period was 12 months. Institutional ethics committee approval was obtained, and all patients provided written informed consent.

The inclusion criteria were adults aged 18 years or older; presence of C3–T1 clinical and radiological pathology at 1–3 intervertebral disc levels (including cervical radiculopathy, myelopathy, symptomatic degenerative disc disease, facet arthropathy, or instability) that had not responded to nonoperative management for a minimum of 6 weeks; and willingness to attend all follow-up visits and imaging. Patients were excluded if they had 4-level or more pathology, trauma, infection, or malignancy. Prior cervical spine surgery was not an exclusion criterion.

### 2.2. Surgical Technique

After patients received prophylactic antibiotics and general anaesthesia with endotracheal intubation in theatre, they were placed supine with their neck in gentle extension. Fluoroscopy was utilised to plan the skin incision. A right transverse skin incision and Smith-Robinson approach to the anterior cervical spine were performed for confirmation of the target disc levels by fluoroscopy. Vertebral body pins were used for in-line disc space distraction. Discectomy, endplate contouring (using a diamond drill, curettes, and rasp), opening of the posterior longitudinal ligament, and decompression of the spinal cord and exiting nerve roots were performed under microscopic illumination. Interbody trials were used to determine the optimal height and width of the impacted cage(s) prior to implant placement.

### 2.3. Interbody Cages

**Figure 1:**
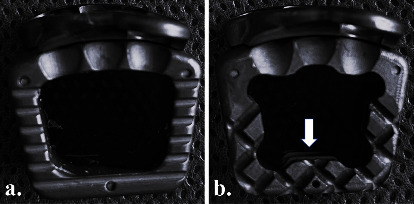
Cages with a molecularly bonded layer of titanium over the entire surface area of the PEEK implant ((a) NM cage) and additional machined porous features (white arrow) on the endplates and within the graft apertures ((b) NMRT cage).

Both types of cages used in this study were machined PEEK with a submicron layer of Ti applied to all exposed surfaces (i.e., NM) (Figure 1). The Shoreline ACS (Anterior Cervical System) cage (SeaSpine, Carlsbad, CA) was the traditional NM cage used in the NM group, whereas the Shoreline Reef Topography™ cage (SeaSpine) was the NM cage with machined porous features at the endplate used in the NMRT group. All cages had 7-degree lordosis and an integrated titanium 2-hole plate-screw fixation (TruProfile Plate; SeaSpine). All cages were filled with the same demineralised allograft fibres (2.5 g, Boost UltraFibres; Australian Biotechnologies, Sydney, Australia).

### 2.4. Clinical Outcomes

Patients were followed up clinically for a minimum of 12 months, according to the usual standard of care at our institution. Patient self-reported outcome measures (PROMs) were evaluated preoperatively (baseline) and at 6 weeks, 3 months, and 6 months postoperatively. PROMs included visual analogue scale (VAS) scores for neck and arm pain, Neck Disability Index (NDI) scores, and 12-Item Short Form Survey (SF-12) scores (both physical and mental components).

### 2.5. Radiographic Outcomes

**Figure 2:**
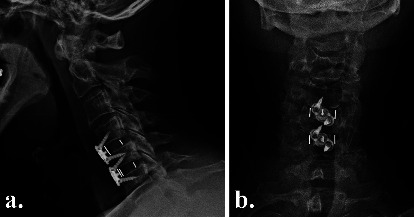
Postoperative (a) lateral and (b) anteroposterior radiographs on day 2, showing NMRT cages with integrated plate/screws at C4/5 and C5/6.

**Figure 3:**
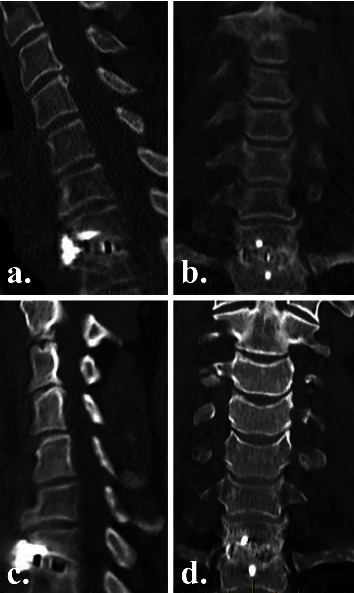
Postoperative computed tomography (a, c) sagittal and (b, d) coronal views of an NM cage group patient at (a, b) 3 months (bridwell grade 2) and (c, d) 6 months (bridwell grade 1) postoperatively, showing progressive interbody fusion at C7/T1.

**Figure 4:**
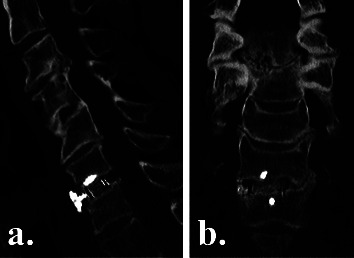
Postoperative computed tomography (a) sagittal and (b) coronal views of an NMRT cage group patient at 3 months postoperatively, showing earlier solid (bridwell grade 1) interbody fusion at C6/7.

**Figure 5:**
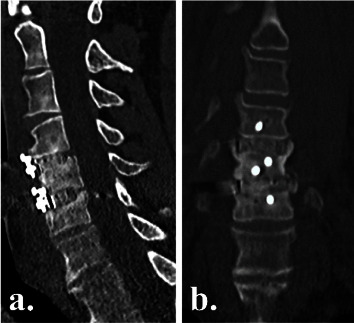
Postoperative computed tomography (a) sagittal and (b) coronal views of an NMRT cage group patient at 3 months postoperatively, showing early solid (bridwell grade 1) fusion at C4/5 and C5/6.

Plain radiographs were obtained on postoperative day 2 to assess instrumentation positioning (Figure 2), and high-definition CT scans were obtained at 3 and 6 months postoperatively to evaluate fusion status (Figure 3), as part of the routine standard of care at our institution. To reduce radiation exposure, no CT scans were performed after confirming interbody fusion. We did not expose patients to more radiation, in the form of CT scans, than the standard of care in Australia. Fusion with new bone formation was assessed using the Bridwell interbody fusion grading system [26, 27], with bridging interbody trabecular bone on coronal and sagittal views graded from 1 to 4 (1 = fused, with trabeculae present; 2 = graft intact, not fully remodelled but no lucency present; 3 = graft intact, with lucency present at the top and bottom of the graft; 4 = fusion absent) (Figures 4 and 5). Grades I or 2 were considered fused, and grades 3 or 4 were deemed not fused. The quality of the fusion was based on the consecutive numerical ranking (Bridwell grades 1–4). Interbody fusion was assessed by independent radiologists from another institution.

### 2.6. Radiation Cost Analysis

Medicare charges for cervical radiographs and CT were obtained from the Australian Government Medicare Benefits Schedule [28]. Radiation dose reports from postoperative CT scans were supplied by the institutional radiology departments and measured as dose length products (DLP, mGy-cm). The DLP was then converted to an effective dose (mSv), accounting for body region and patient age [29].

### 2.7. Complications

Complications were identified during hospitalisation and after discharge for final clinical follow-up. They included airway compromise, neurologic deficit, dysphonia, dysphagia, surgical-site infection, cage subsidence, reoperation, and mortality.

### 2.8. Ethical Statement

This was a review of cases collected under a standard privacy disclosure to patients that their information will be used for ongoing evaluation of outcomes and their identity will be protected in any publication arising from this. The project was reviewed by an independent expert in Human Research Ethics and classified as a low-risk research project in accordance with section 5.1.19 of the National Statement on Ethical Conduct in Human Research (2007). Institutional approval was granted by Epworth HealthCare (EH2020-514). The authors are accountable for all aspects of the work in ensuring that questions related to the accuracy or integrity of any part of the work are appropriately investigated and resolved.

### 2.9. Statistical Methods

The sample size was calculated using G^*∗*^Power (Heinrich-Heine-Universitat, Dusseldorf, Germany) with a medium effect size of 0.5, alpha of 0.05, and power of 0.80. Mean, standard deviation, and 95% confidence interval (CI) were calculated for subject demographic, VAS pain score, NDI, and SF-12 data. A Pearson's R linear correlation test was performed to assess the relationship between bony fusion and clinical outcomes, with a significance of >0.7. VAS Neck, VAS Arm, Mental SF-12 and Physical SF-12 were analysed for correlation via a Pearson's correlation coefficient test. Statistical analyses were performed using Microsoft Excel (Microsoft Corp, Seattle, WA) and Stata (Version SE 17.0, Stata Corp, College Station, TX) and included paired *t*-tests, independent samples *t*-tests, and Fisher exact tests. Statistical significance was set at *p* < 0.05.

## 3. Results

### 3.1. Patient Characteristics

A total of 89 patients were included in the study over a follow-up range of 12 to 34 months (mean: 14 months). Their mean age was 62 ± 10.5 years (95% CI 59.8–64.2), and 46 (52%) were male (Table 1). Forty-one patients received NM cages and 48 patients received NMRT cages. The most common presenting pathologies were radiculopathy (*n* = 57; 64%) and myelopathy (*n* = 22; 25%). There were no significant differences in age (*p*=0.37), sex (*p*=0.27), or presenting pathology (*p*=0.76) between the NM and NMRT groups.

### 3.2. Surgical Data

Sixty-eight patients underwent 1-level ACDF, 15 patients underwent 2-level ACDF, and 6 patients underwent 3-level ACDF. A total of 116 operative levels were treated, with the majority being C6/7 (*n* = 39; 34%) and C5/6 (*n* = 37; 32%). There was no difference in levels treated between the NM and NMRT groups (Table 1).

### 3.3. Clinical Outcomes

All clinical outcomes exhibited significant improvement from baseline to 6-month follow-up in both the NM and NMRT groups (Table 2). The improvement was greater in the NMRT group than in the NM group, although the difference between groups was not statistically significant (53% vs 45%, *p*=0.65). By last follow-up, mean neck and arm VAS pain scores improved 73% and 78%, respectively, in the NMRT group and 55% and 59%, respectively, in the NM group, but the improvements did not differ significantly between groups (*p*=0.88; *p*=0.76). NDI improved 76% in the NMRT group and 70% in the NM group, also with no significant difference in improvement between groups (*p*=0.85). Likewise, improvement in quality of life trended toward more improvement in the NMRT group than in the NM group for both physical SF-12 scores (29% vs 23%, *p*=0.79) and mental SF-12 scores (16% vs 13%, *p*=0.33).

### 3.4. Radiographic Outcomes

**Figure 6:**
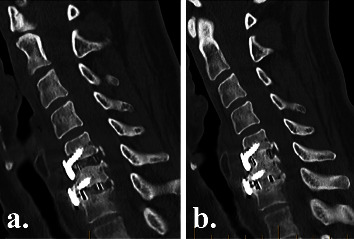
Postoperative computed tomography sagittal views of NM cage group patient at (a) 3 months showing incomplete (bridwell grade 4) fusion and at (b) 9 months, showing consolidated solid (bridwell grade 1) fusion at C5/6 and C6/7.

By 3 months postoperatively, CT fusion rates were significantly higher in the NMRT group than in the NM group (79% vs 56%, *p*=0.02) (Figures 3–6). However, by 6 months postoperatively, there was no significant difference in fusion rates between the NMRT and NM groups (83% vs 78%, *p*=0.69). Thus, fusion occurred earlier with NMRT, but fusion rates were similar between groups by 6 months after surgery.

On qualitative assessment (Bridwell grades 1–4) of new bone formation at 6 months postoperatively, there was a trend toward better fusion quality in the NMRT group (mean score, 1.4 ± 0.7) than in the NM group (mean score, 1.8 ± 1.0). This indicates that the NMRT group exhibited 22% greater improvement in fusion quality compared with the NM group, although this was not statistically significant (*p*=0.08).

### 3.5. Radiation Cost Analysis

The Medicare charge for the day 2 cervical radiograph was A$68.75, and the postoperative CT scan was A$245.80 [28]. The mean postoperative CT DLP was 467.5 mGycm (range 398–555), which is equivalent to an effective dose of 2.76 mSv (range 2.35–3.27).

### 3.6. Clinical Outcome Correlation

NMRT patients VAS Neck scores demonstrated significant improvement from baseline 6-month follow up (*p*=0.0004) and an *R*^2^ correlation of 0.77. NM patients exhibited a significant improvement in VAS Neck scores from baseline to 6-month follow up (*p*=0.001) and an *R*^2^ correlation of 0.75.

NMRT patients VAS Arm demonstrated significant improvement from baseline 6-month follow up (*p*=0.0002) and an *R*^2^ correlation of 0.82. NM patients exhibited a significant improvement in VAS Arm scores from baseline to 6-month follow up (*p*=0.008) and an *R*^2^ correlation of 0.70.

NMRT patients physical SF-12 scores demonstrated significant improvement from baseline 6-month follow up (*p*=0.002) and an *R*^2^ correlation of 0.88. NM patients exhibited a significant improvement in Physical SF-12 scores from baseline to 6-month follow up (*p*=0.003) and an *R*^2^ correlation of 0.88.

NMRT patients mental SF-12 scores demonstrated significant improvement from baseline 6-month follow-up (*p*=0.001) and an *R*^2^ correlation of 0.70. NM patients exhibited a significant improvement in Mental SF-12 scores from baseline to 6-month follow-up (*p*=0.001) and an *R*^2^ correlation of 0.78.

### 3.7. Complications

The total complication rate was 5.6% (5/89) for the final follow-up. There was no difference in approach-related morbidity between the NM group (2/41; 4.9%) and the NMRT group (3/48; 6.3%). No subsidence, revision surgery at the index levels, adjacent level surgery, or return to the operating room occurred in either group.

## 4. Discussion

Fusion rates in ACDF are high. A systematic literature review of 146 articles comprising 10,208 patients reported bony fusion in over 90% of patients at 12- to 24-month follow-up [9]. Nonunion after ACDF can lead to ongoing pain, a neurologic deficit, and further surgery [11]. The aetiology of nonunion is multifactorial and includes patient factors (diabetes, smoking), surgical technique, operative level, type of implant (cage type, presence or absence of plating), and type of graft [13]. True rates of radiographic nonunion and symptomatic nonunion are difficult to determine since many patients with good results do not undergo postoperative imaging, clinical follow-up, or both.

Cages are used in almost 70% of ACDF surgeries, most commonly PEEK cages (46%), followed by Ti cages (17%) [9]. We investigated the use of cages with a molecularly bonded layer of Ti on the entire surface area of the PEEK implant, including the endplates and throughout the graft apertures, to optimise bone on-growth at the surface of the NM cages and in-growth into the apertures of the NMRT cages. These cages retain the benefits of PEEK implants, such as biocompatibility, a modulus of elasticity similar to that of bone, and radiographic visibility for postoperative imaging. Both the NM and NMRT cages are manufactured by traditional techniques and have a fixed cost (AUD$4534) as set by the Australian healthcare system regulatory body (the Therapeutic Goods Administration).

In ACDF, the addition of anterior plates results in higher fusion rates compared with stand-alone implants, based on systematic review and meta-analysis [5, 9]. There is no difference in clinical or radiographic outcomes between using a combined plate-cage construct or a separate anterior buttress plate and cage [30]. We used low-profile integrated 2-hole plate-screw fixation rather than separate anterior buttress plating for ease of use, as well as to shorten the operation time and decrease the risk of dysphagia, subsidence, and adjacent segment disease [6, 30]. There was no significant difference in complication rates between the NM and NMRT groups (4.9% vs 6.3%), and these rates were similar to the 2.4% to 7% complication rates of ACDF reported in previous systematic reviews and meta-analyses [31–33].

Cadaveric demineralised allograft fibres were used to fill our study cages to avoid iliac crest bone harvesting and provide osteoinductive and osteogenic properties with higher fusion rates than osteoconductive synthetic bone substitutes [34, 35]. The use of demineralised fibres ensured that any bony interbody opacities represented true new bone formation originating from the fibres, not radiopacities from autografts, allograft blocks, or synthetics.

In recent systematic literature reviews, cervical interbody fusion was assessed by qualitative visualisation of trabecular bridging on plain anteroposterior and lateral radiographs in 44%–79% of studies, by quantitative determination of the absence of motion on dynamic x-rays in 35%–56% of studies, and by visualisation of continuous bridging bone on CT scans in 18%–54% of studies [9, 11]. The effective radiation dose exposure for static cervical radiographs is 0.2 mSv, and a total of 0.4 mSv radiation is required for dynamic views [36, 37]. We used approximately 2.8 mSv for CT imaging in the current study. Use of flexion/extension x-rays to evaluate interspinous process motion <1 mm and Cobb angle change <2 degrees between adjacent fused vertebrae is superior to assessment with plain radiographs, but dynamic x-rays are difficult to obtain in elderly patients with advanced spondylosis [11, 38]. We used fine-cut CT to assess fusion grade, given its superiority for assessing both intragraft and extragraft trabecular bridging bone on reconstructed coronal and sagittal views, compared with plain films [12, 38]. The Bridwell grading system has high interobserver reliability [27, 39]. Further CT indicators of nonunion include peri-instrumentation halo signs, which enhance the sensitivity and specificity of pseudoarthrosis detection [11]. Our study found that CT was associated with an approximately 10 times higher radiation dose and 3.5 times higher cost than radiographs.

Fusion rates over time have been reported as approximately 50%, 75%, and 90% at 3, 6, and 12 months following ACDF [9, 10]. In the current study, we found a significantly earlier fusion rate with NMRT cages, compared with NM cages (79% vs 56%), at 3 months. Fusion rates were similar at 6 months (83% vs 78%), although the quality of fusion at 6 months tended to be better in the NMRT group, with a 22% higher Bridwell score.

Following ACDF surgery, many surgeons assess patients at 6 weeks postoperatively, and if satisfactory clinical results are observed, no further review or imaging is scheduled. Ongoing neck pain, disability, or functional impairment following ACDF suggest the possibility of nonunion. An important goal of surgeons and patients is faster recovery, with earlier stability and cage integration. The superior early fusion rates (at 3 months) in the NMRT group compared to the NM group suggest that cage aperture in-growth fusion is beneficial. Hence, earlier bone integration may lead to earlier biomechanical stability, leading to earlier fusion.

We found that as fusion rates improved at 3 and then 6 months postoperatively with both NM and NMRT cages, so did the PROMs. Both the NM and NMRT groups showed significant improvements in VAS neck and arm pain scores, NDI, and SF-12 from preoperatively to 6 weeks, 3 months, and then 6 months postoperatively. Nevertheless, there were no significant differences in improvement between groups. Few studies have correlated clinical outcomes with bony fusion at different time points. Most studies have found no association between fusion and clinical results [14, 15, 17, 19]. However, some studies have reported a significant correlation between successful fusion and superior clinical outcomes. In a retrospective analysis of PEEK and polymethyl methacrylate (PMMA) cages, Klinger et al. [16] found that patients with CT fusion had significantly better SF-36 (but not NDI or VAS) scores than those without fusion. Wright and Eisenstein [20] prospectively studied patients with autografts and found that the absence of fusion on dynamic x-rays was correlated with higher VAS scores for neck pain, but not arm pain. Similarly, Ouchida et al. [40] reported that patients with solid fusion by functional CT at 6 months had lower VAS neck pain scores than those without fusion. In a prospective study of Ti cages, Schroder et al. [18] found that fusion assessed by plain x-rays was correlated with excellent and good results using Odom's criteria, whereas the absence of fusion was correlated with satisfactory and poor results.

We performed CT scans 3 months after surgery to assess the early fusion process instead of the standard 12-month postoperative scan. We did not expose patients to more radiation than the standard of care in Australia. We showed CT evaluation at this early timepoint after surgery, when interbody fusion is usually not considered consolidated in most patients, which enabled us to correlate bony fusion with clinical outcomes in the early recovery period and at our later 6-month study timepoint. As fusion was observed at 3 months in the majority (79%) of patients who received NMRT cages, this is of benefit to patients, permitting earlier return to work and sensible activity at this early postoperative timepoint instead of waiting for a standard 12-month follow-up CT scan. Earlier physical activity has been shown to have benefits, such as pain reduction [41]. We therefore suggest obtaining x-rays on postoperative day 2 for assessment of cage, plate, and screw placement to provide reassurance for the surgeon and patient and obtaining a CT scan at 3 months postoperatively, which is sufficient in almost 80% of patients undergoing NMRT cage insertion to avoid additional CT radiation. The traditional 12-month postoperative CT for ACDF may not be warranted with either NM or NMRT cages, as in most patients, it will merely confirm the presence of more consolidated interbody bone.

The strengths of this study include its prospective, consecutive design, and the use of a consistent surgical technique, which was performed by two senior surgeons who were very experienced in ACDF procedures. In both treatment groups, the cages were filled with the same demineralised allograft fibres to ensure that any new interbody bone formation was from the fibres (not radiopacities introduced from autograft, allograft blocks, or synthetics) and to ensure that the only variable in the study design was the type of cage. Both the NMRT and NM cages with and without machined porous features are manufactured for a similar cost. Thin-section early CT assessment confirmed interbody fusion, despite higher costs and radiation exposure, compared with plain static and flexion-extension radiographs. Serial postoperative PROMs and CT showed a good correlation between clinical outcomes and earlier bony fusion at our study timepoints.

The limitations of this study included the relatively small cohort sizes of the two cage groups. The study was not randomised but undertaken on consecutive patients. The intermediate duration of follow-up (6 months of radiographic and a minimum of 12 months clinical) precluded assessment of late subsidence, pseudoarthrosis, or reoperation rates. Future studies with randomised larger cohorts and longer follow-up will provide further information.

With both NM cages and NMRT cages, serial improvements in postoperative clinical outcomes were associated with the progression of fusion on CT images. However, NMRT cages demonstrated a significantly higher fusion rate at 3 months and a trend toward superior quality of fusion (Bridwell grade) at 6 months after ACDF, compared with NM cages. The earlier stability of NMRT cages appears to indicate earlier cage integration. An early 3-month postoperative CT was adequate for detection of fusion in almost 80% of patients undergoing ACDF with an NMRT cage. The traditional 12-month postoperative CT for ACDF may not be justified when using either NM or NMRT cages; in most patients, it will merely confirm the presence of more consolidated interbody bone.

## Figures and Tables

**Table 1:** Patient demographics and operative levels.

Characteristic	Total	*p* value
Total patients	89	
NM patients	41	0.20
NMRT patients	48	
Males	46	0.27
Females	43	
Mean age (years)	62 ± 10.5 (95% CI 59.8–64.2)	0.37
Age range (years)	42–80	
Presenting pathology		
NM group		
Disk prolapse	10	
Foraminal stenosis	15	
Cord compression	13	
Degenerative disk disease	3	
		0.76
NMRT		
Disk prolapse	16	
Foraminal stenosis	16	
Cord compression	9	
Degenerative disk disease	7	
Surgical Levels		
1-level ACDF	68	
2-level ACDF	15	
3-level ACDF	6	
Cervical Levels		
C3/4	10	
C4/5	19	
C5/6	37	
C6/7	39	
C7/T1	11	

Data are number, mean ± standard deviation, or range. CI, confidence interval.

**Table 2:** Patient reported outcome scores.

Cohort	Preop neck VAS	6-week neck VAS	3-month neck VAS	6-month neck VAS	Preop vs 6-month scores (*p* value)	Change (%)	NM vs NMRT (*p* value)
NM	4.9 ± 3.0	2.7 ± 2.2	1.7 ± 2.2	1.7 ± 2.5	0.00	55.0	0.8
	(95% CI 3.98–5.82)	(95% CI 2.03–3.37)	(95% CI 1.03–2.37)	(95% CI 0.935–2.46)			
NMRT	5.3 ± 2.7	2.1 ± 2.3	1.4 ± 1.9	1.4 ± 2.2	0.00	73.0	
	(95% CI 4.54–6.06)	(95% CI 1.45–2.75)	(95% CI 0.862–1.94)	(95% CI 0.778–2.02)			

	Preop arm VASs	6-week arm VAS	3-month arm VAS	6-month neck			

NM	6.6 ± 2.9	2.6 ± 2.4	1.6 ± 2.1	1.1 ± 1.7	0.00	59.0	0.76
	(95% CI 5.71–7.49)	(95% CI 1.87–3.33)	(95% CI 0.97–2.24)	(95% CI 0.58–1.62)			
NMRT	5.5 ± 2.9	1.7 ± 2.2	1.3 ± 2.2	1.2 ± 1.8	0.0002	78.0	
	(95% CI 4.68–6.32)	(95% CI 1.08–2.32)	(95% CI 0.678–1.92)	(95% CI 0.961–1.71)			

	Preop NDI	6-week NDI	3-month NDI	6-month NDI			

NM	30.1 ± 16.3	17.5 ± 16.1	9.9 ± 8.8	7.3 ± 8.6	0.0009	76.0	0.85
	(95% CI 27.1–36.9)	(95% CI 12.6–22.4)	(95% CI 7.21–12.6)	(95% CI 4.67–9.93)			
NMRT	32.0 ± 17.4	1.81 ± 17.0	10.8 ± 11.6	9.3 ± 14.4	0.004	70.0	
	(95% CI 27.1–36.9)	(95% CI 13.3–22.9)	(95% CI 7.52–14.1)	(95% CI 5.23–13.4)			

	Preop physical SF-12	6-week physical SF-12	3-month physical SF-12	6-month physical SF-12			

NM	36.7 ± 9.1	43.6 ± 9.9	46.6 ± 7.3	47.7 ± 6.3	0.003	23.0	0.79
	(95% CI 33.9–39.5)	(95% CI 40.6–46.6)	(95% CI 44.4–48.8)	(95% CI 45.8–49.6)			
NMRT	33.4 ± 10.3	43.7 ± 10.8	46.0 ± 10.9	47.2 ± 9.7	0.002	29.0	
	(95% CI 30.5–36.3)	(95% CI 40.6–46.8)	(95% CI 43–49)	(95% CI 44.3–49.7)			

	Preop mental SF-12	6-week mental SF-12	3-month mental SF-12	6-month mental SF-12			

NM	42.9 ± 10.3	49.3 ± 8.3	49.8 ± 6.7	50.2 ± 5.9	0.001	13.0	0.33
	(95% CI 40–45.8)	(95% CI 46.8–51.8)	(95% CI 47.8–51.8)	(95% CI 48.4–52)			
NMRT	45.0 ± 10.8	51.3 ± 7.96	52.9 ± 7.8	53.4 ± 8.6	0.001	16.0	
	(95% CI 40–45.8)	(95% CI 49–53.5)	(95% CI 50.7–55.1)	(95% CI 51–55.8)			

CI, confidence interval; NDI, Neck Disability Index; SF-12, 12-Item -Short Form Survey; VAS, visual analogue scale.

## Data Availability

Institutional ethics approval was granted to collect cohort data. The cohort data used to support the findings of this study are restricted by the institution in order to protect patient confidentiality. The cohort data used to support the findings of this study are available for researchers who meet the criteria for access to confidential data from the corresponding author upon request.

## References

[1] Saifi C., Fein A. W., Cazzulino A. (2018). Trends in resource utilization and rate of cervical disc arthroplasty and anterior cervical discectomy and fusion throughout the United States from 2006 to 2013. *The Spine Journal*.

[2] Smith G. W., Robinson R. A. (1958). The treatment of certain cervical-spine disorders by anterior removal of the intervertebral disc and interbody fusion. *Journal of Bone and Joint Surgery*.

[3] Walsh W. R., Pelletier M., Wills D., Wang T., Bannigan S., Vizesi F. (2020). Undercut macrostructure topography on and within an interbody cage improves biomechanical stability and interbody fusion. *The Spine Journal*.

[4] Suess O., Schomaker M., Cabraja M., Danne M., Kombos T., Hanna M. (2017). Empty polyetheretherketone (PEEK) cages in anterior cervical diskectomy and fusion (ACDF) show slow radiographic fusion that reduces clinical improvement: results from the prospective multicenter “PIERCE-PEEK” study “PIERCE-PEEK” study. *Patient Safety in Surgery*.

[5] Oliver J. D., Goncalves S., Kerezoudis P. (2018). Comparison of outcomes for anterior cervical discectomy and fusion with and without anterior plate fixation: a systematic review and meta-analysis. *Spine*.

[6] Zhao Y., Yang S., Huo Y., Li Z., Yang D., Ding W. (2020). Locking stand-alone cage versus anterior plate construct in anterior cervical discectomy and fusion: a systematic review and meta-analysis based on randomized controlled trials. *European Spine Journal*.

[7] Buyuk A. F., Onyekwelu I., Gaffney C. J. (2020). Symptomatic pseudarthrosis requiring revision surgery after 1- or 2-level ACDF with plating: peek versus allograft. *J Spine Surg*.

[8] Iunes E. A., Barletta E. A., Belsuzarri T. A. B. (2020). Pseudarthrosis in anterior cervical discectomy and fusion with a self-locking, stand-alone cage filled with hydroxyapatite: a retrospective study with clinical and radiological outcomes of 98 levels with a minimum 2-year follow-up. *Journal of Neurosurgery: Spine*.

[9] Noordhoek I., Koning M. T., Vleggeert-Lankamp C. L. A. (2019). Evaluation of bony fusion after anterior cervical discectomy: a systematic literature review. *European Spine Journal*.

[10] Park D. K., Rhee J. M., Kim S. S., Enyo Y., Yoshiok K. (2015). Do CT scans overestimate the fusion rate after anterior cervical discectomy and fusion?. *Journal of Spinal Disorders & Techniques*.

[11] Lin W., Ha A., Boddapati V., Yuan W., Riew K. D. (2018). Diagnosing pseudoarthrosis after anterior cervical discectomy and fusion. *Neurospine*.

[12] Riew K. D., Yang J. J., Chang D. G. (2019). What is the most accurate radiographic criterion to determine anterior cervical fusion?. *The Spine Journal*.

[13] Oshina M., Oshima Y., Tanaka S., Riew K. D. (2018). Radiological fusion criteria of postoperative anterior cervical discectomy and fusion: a systematic review. *Global Spine Journal*.

[14] Cabraja M., Oezdemir S., Koeppen D., Kroppenstedt S. (2012). Anterior cervical discectomy and fusion: comparison of titanium and polyetheretherketone cages. *BMC Musculoskeletal Disorders*.

[15] Choi M. K., Kim S. B., Park C. K., Kim S. M. (2016). Comparison of the clinical and radiologic outcomes obtained with single- versus two-level anterior cervical decompression and fusion using stand-alone PEEK cages filled with allograft. *Acta Neurochirurgica*.

[16] Klingler J. H., Krüger M. T., Sircar R. (2014). PEEK cages versus PMMA spacers in anterior cervical discectomy: comparison of fusion, subsidence, sagittal alignment, and clinical outcome with a minimum 1-year follow-up. *The Scientific World Journal*.

[17] Park J. I., Cho D. C., Kim K. T., Sung J. K. (2013). Anterior cervical discectomy and fusion using a stand-alone polyetheretherketone cage packed with local autobone: assessment of bone fusion and subsidence. *J Korean Neurosurg Soc*.

[18] Schröder J., Grosse-Dresselhaus F., Schul C., Wassmann H. (2006). Anterior cervical spinal fusion with the Intromed ZWE System: preliminary experience. *Neurosurgical Review*.

[19] Shiban E., Gapon K., Wostrack M., Meyer B., Lehmberg J. (2016). Clinical and radiological outcome after anterior cervical discectomy and fusion with stand-alone empty polyetheretherketone (PEEK) cages. *Acta Neurochirurgica*.

[20] Wright I. P., Eisenstein S. M. (2007). Anterior cervical discectomy and fusion without instrumentation. *Spine*.

[21] Seaman S., Kerezoudis P., Bydon M., Torner J. C., Hitchon P. W. (2017). Titanium vs. polyetheretherketone (PEEK) interbody fusion: meta-analysis and review of the literature. *Journal of Clinical Neuroscience*.

[22] Rao P. J., Pelletier M. H., Walsh W. R., Mobbs R. J. (2014). Spine interbody implants: material selection and modification, functionalization and bioactivation of surfaces to improve osseointegration. *Orthopaedic Surgery*.

[23] Torstrick F. B., Klosterhoff B. S., Westerlund L. E. (2018). Impaction durability of porous polyether-ether-ketone (PEEK) and titanium-coated PEEK interbody fusion devices. *The Spine Journal*.

[24] Walsh W. R., Pelletier M. H., Christou C., He J., Vizesi F., Boden S. D. (2018). The in vivo response to a novel Ti coating compared with polyether ether ketone: evaluation of the periphery and inner surfaces of an implant. *The Spine Journal*.

[25] Fogel G., Martin N., Lynch K. (2022). Subsidence and fusion performance of a 3D-printed porous interbody cage with stress-optimized body lattice and microporous endplates-a comprehensive mechanical and biological analysis. *The Spine Journal*.

[26] Bridwell K. H., Lenke L. G., McEnery K. W., Baldus C., Blanke K. (1995). Anterior fresh frozen structural allografts in the thoracic and lumbar spine: do they work if combined with posterior fusion and instrumentation in adult patients with kyphosis or anterior column defects. *Spine*.

[27] Kim S. Y., Park K. S., Jung S. S. (2012). An early comparative analysis of the use of autograft versus allograft in anterior cervical discectomy and fusion. *Korean J Spine*.

[28] AgdoH (2022). Medicare benefits Schedule: MBS online. http://www9.health.gov.au/mbs/search.cfm.

[29] Nett B. (2022). Simple calculator for effective dose in CT (DLP-> eff dose). Radiologic technologist’s guide to effective dose (mSv) in CT from dose length product (mGy cm) how radiology works: how radiology works. https://howradiologyworks.com/dlp-calculator/.

[30] Nambiar M., Phan K., Cunningham J. E., Yang Y., Turner P. L., Mobbs R. (2017). Locking stand-alone cages versus anterior plate constructs in single-level fusion for degenerative cervical disease: a systematic review and meta-analysis. *European Spine Journal*.

[31] Liu W. J., Hu L., Chou P. H., Wang J. W., Kan W. S. (2016). Comparison of anterior cervical discectomy and fusion versus posterior cervical foraminotomy in the treatment of cervical radiculopathy: a systematic review. *Orthopaedic Surgery*.

[32] Rogerson A., Aidlen J., Mason A., Pierce A., Tybor D., Salzler M. J. (2021). Predictors of inpatient morbidity and mortality after 1- and 2-level anterior cervical diskectomy and fusion based on the national inpatient sample database from 2006 through 2010. *Orthopedics*.

[33] Wichmann T. O., Bech-Azeddine R., Norling A. L., Einarsson H. B., Rasmussen M. M. (2021). Comparison of outcomes and complications between one- and two-level anterior cervical discectomy and fusion: a population-based study of 410 patients. *British Journal of Neurosurgery*.

[34] Buser Z., Brodke D. S., Youssef J. A. (2016). Synthetic bone graft versus autograft or allograft for spinal fusion: a systematic review. *Journal of Neurosurgery: Spine*.

[35] Dimitriou R., Mataliotakis G. I., Angoules A. G., Kanakaris N. K., Giannoudis P. V. (2011). Complications following autologous bone graft harvesting from the iliac crest and using the RIA: a systematic review. *Injury*.

[36] Oakley P. A., Harrison D. E. (2018). Radiophobia: 7 reasons why radiography used in spine and posture rehabilitation should not Be feared or avoided. *Dose-Response*.

[37] Simpson A. K., Whang P. G., Jonisch A., Haims A., Grauer J. N. (2008). The radiation exposure associated with cervical and lumbar spine radiographs. *Journal of Spinal Disorders & Techniques*.

[38] Rhee J. M., Chapman J. R., Norvell D. C., Smith J., Sherry N. A., Riew K. D. (2015). Radiological determination of postoperative cervical fusion: a systematic review. *Spine*.

[39] Tan G. H., Goss B. G., Thorpe P. J., Williams R. P. (2007). CT-based classification of long spinal allograft fusion. *European Spine Journal*.

[40] Ouchida J., Yukawa Y., Ito K. (2015). Functional computed tomography scanning for evaluating fusion status after anterior cervical decompression fusion. *European Spine Journal*.

[41] Coronado R. A., Devin C. J., Pennings J. S. (2020). Early self-directed home exercise program after anterior cervical discectomy and fusion: a pilot study. *Spine*.

